# Mechanical Performance of FDM-Printed PLA Joined by Portable Friction Stir Welding: Influence of Infill Density and Tool Pin Geometry

**DOI:** 10.3390/polym18091013

**Published:** 2026-04-22

**Authors:** Juan Antonio Almazán, Miguel Ángel Almazán, Marta M. Marín, Amabel García-Domínguez, Eva María Rubio

**Affiliations:** 1 Department of Mechanical and Mining Engineering, A3 Building—Campus Las Lagunillas, University of Jaen, s/n, 23071 Jaen, Spain; jalmazan@ujaen.es; 2 Teaching and Research Laboratories of the Central Research Support Services (SCAI), A2 Building—Campus Las Lagunillas, University of Jaen, s/n, 23071 Jaen, Spain; aalmazan@ujaen.es; 3 Department of Manufacturing Engineering, Industrial Engineering School, Universidad Nacional de Educación a Distancia (UNED), St/Juan del Rosal 12, 28040 Madrid, Spain; mmarin@ind.uned.es (M.M.M.); agarcia@ind.uned.es (A.G.-D.)

**Keywords:** Friction Stir Welding (FSW), fused deposition modelling (FDM), poly(lactic acid) (PLA), portable welding device, infill density, tool pin geometry, tensile properties, additive manufacturing

## Abstract

This study evaluates the mechanical performance of FDM-printed poly(lactic acid) (PLA) structures joined using a portable Friction Stir Welding (FSW) device. A non-destructive optical band method was employed to assess weld homogeneity and material flow consistency. The influence of substrate infill density (15% and 100%) and tool pin geometry (cylindrical and truncated conical) was systematically analyzed. Results indicate that substrate density is the primary determinant of joint integrity; 100% infill specimens demonstrated superior structural homogeneity and consistent intensity profiles, whereas 15% infill specimens exhibited significant intensity fluctuations and poor consolidation, even with the addition of filler material. The mechanical evaluation revealed that the use of a tool pin is essential for effective load transfer, as specimens welded without internal agitation achieved only baseline tensile strengths of approximately 4 MPa. Among the pin-driven configurations, the cylindrical geometry outperformed the truncated conical design, reaching a peak tensile stress of 8.02 ± 1.42 MPa, corresponding to a joint efficiency of 27% relative to the 100% infill base material, compared to 6.25 ± 1.43 MPa. This performance gap is attributed to the cylindrical pin’s ability to maintain higher shear rates and more uniform pressure distribution at the weld root. These findings demonstrate the feasibility of portable FSW for structural joining of additively manufactured polymers and establish critical processing parameters for the optimization of portable FSW in engineering applications.

## 1. Introduction

The advancement of joining technologies has been essential in the evolution of industrial manufacturing, particularly with the integration of novel materials produced via additive manufacturing (AM). While traditional joining methods, such as mechanical fastening and adhesive bonding, offer feasible solutions, permanent welding techniques are often essential for ensuring superior structural durability and integrity [1]. Friction Stir Welding (FSW) represents a significant alternative in solid-state welding. Originally developed for aluminum alloys, its application has expanded significantly to include other metals [2], polymers and AM-fabricated components, offering a way to join materials below their melting point to avoid thermal degradation [1,3].

Additive manufacturing, specifically Fused Deposition Modeling (FDM), enables the construction of complex geometries with high material efficiency. It is one of the most cost-effective and accessible techniques, considering equipment, operational, and material costs [4]. Furthermore, it allows for the use of a wide range of polymers, including biodegradable thermoplastics such as poly(lactic acid) (PLA) [5,6,7]. However, the mechanical performance of FDM parts depends heavily on printing parameters [8]. For instance, Yıldız et al. [9] demonstrated the mechanical influence of the stacking sequences and infill patterns in FDM structures. Furthermore, Ergene et al. [10] showed that infill rates and environmental factors directly influence the wear performance of printed components. Also, FDM parts inherently suffer from mechanical anisotropy, as the interlayer bond is typically the weakest point [11,12]. PLA is the most widely used polymer in FDM, both at domestic and industrial level, due to its low cost, minimal warping, and ease of processing [13,14]. This makes the study of its weldability particularly relevant. PLA offers higher tensile strength and dimensional accuracy than other alternatives such as ABS or PETG in FDM conditions [15,16]. Regarding its thermal properties, Tg around 60–65 °C and Tm around 160–170 °C [17] provide an adequate processing window for FSW, enabling plasticization without degradation [18]. Its low thermal shrinkage, biodegradability, and proven biocompatibility [19] further support its selection as the base material in this work.

Joining FDM parts is often necessary due to inherent limitations in build volume. However, conventional joining methods present several drawbacks. Adhesive bonding, although widely used, often results in brittle joints, and it has long curing times depending on the thickness and surface preparation [20], limiting its structural reliability [21]. Similarly, mechanical fastening methods, such as screws, introduce stress concentrations that can exacerbate failure in the inherently anisotropic structure of FDM-printed polymers [22]. Laser welding is limited to transparent and absorbing substrates [23]. Other techniques such as ultrasonic and resistance welding have shown promising joint strengths [24], but rely on specialized equipment that is difficult to adapt to portable or manual tooling configurations. In this context, friction stir welding (FSW) has emerged as a promising alternative. Being a solid-state process, FSW avoids bulk melting and minimizes defects such as porosity [25] while the intense plastic deformation and molecular interdiffusion it induces effectively consolidate the layered FDM architecture into a monolithic joint, overcoming the inherent anisotropy described above [26,27]. Despite these advantages, the application of FSW to polymers remains challenging due to their low thermal conductivity and narrow processing window, which require precise control of heat input [22].

The main process parameters in Friction Stir Welding (FSW), namely tool rotational speed, traverse (welding) speed, and tool tilt angle, play a decisive role in heat generation, material flow behavior, microstructural development, and ultimately the mechanical performance of the welded joint. Among these variables, rotational speed directly influences frictional heat input. Higher rotational speeds generally promote increased heat generation and enhanced plasticization of the material, facilitating improved mixing and consolidation within the stir zone. However, excessive heat input may lead to microstructural coarsening, softening, or even defect formation if optimal thermal conditions are exceeded [28,29].

Traverse speed, on the other hand, controls the interaction time between the rotating tool and the workpiece. When traverse speed is too high, the reduced thermal exposure can result in insufficient plastic flow and incomplete consolidation, potentially lowering joint strength. In contrast, lower traverse speeds increase heat input per unit length, which can enhance metallurgical bonding, although excessive heat accumulation must still be avoided [30,31].

The tool tilt angle also contributes significantly to weld quality. A slight backward tilt, typically between 1° and 3° in metallic FSW applications, improves forging action behind the tool shoulder, promoting better material consolidation and reducing the likelihood of internal voids or tunnel defects. Nevertheless, excessive tilt angles may disturb the stability of material flow and adversely affect joint integrity [32,33].

Such relationships between processing parameters, heat input, and mechanical performance have been widely documented in aluminum alloys and stainless steels, and similar trends have been reported in polymer FSW [21], where careful optimization of thermal and mechanical conditions is essential to achieving high joint efficiency and structural soundness.

The role of tool pin geometry remains a subject of ongoing debate, as contradictory findings have been reported regarding the relative performance of cylindrical and conical pins. While cylindrical pins have been associated with superior mechanical behavior in materials like HDPE, conical pins have demonstrated improved mechanical behavior in ABS, indicating that the effectiveness of a given geometry is highly material-dependent and strongly influenced by the rheological and flow characteristics.

In polymeric materials, the low thermal conductivity and marked viscoelastic response make tight control of the processing parameters essential. Inadequate combinations of rotational and traverse speeds can promote thermal degradation, void formation, or limited molecular interdiffusion at the interface, leading to reduced joint strength and welding efficiency [21].

While prior research has explored FSW in various polymers, such as HDPE, PP, ABS, PC, PMMA, nylon or PEEK [25], most studies rely on rigid CNC-based systems under controlled laboratory conditions [34,35], leaving the feasibility of flexible, manual solutions under-researched. In particular, the mechanical performance of FDM-printed PLA structures joined using portable or semi-manual FSW devices has received limited attention, despite the fact that process stability and consolidation mechanisms may differ significantly from industrial setups. Furthermore, the combined effect of infill density and tool pin geometry on joint integrity has not been systematically quantified. Establishing a clear correlation between the tensile response and the resulting weld microstructure is essential for process optimization and practical implementation in repair and hybrid manufacturing scenarios. This study addresses this gap through a rigorous mechanical characterization, focusing on the tensile behavior of FDM-printed PLA specimens joined with a custom-designed manual tool. By analyzing stress–strain profiles and failure modes across different configurations, this research identifies the optimal processing window for high-strength joints. These mechanical findings are further validated through the optical homogeneity analysis, providing a dual-layered approach to quality assessment. Ultimately, this work supports the potential development of hybrid manufacturing strategies for the aerospace, medical, and automotive sectors [36].

## 2. Materials and Methods

**Figure 1:**
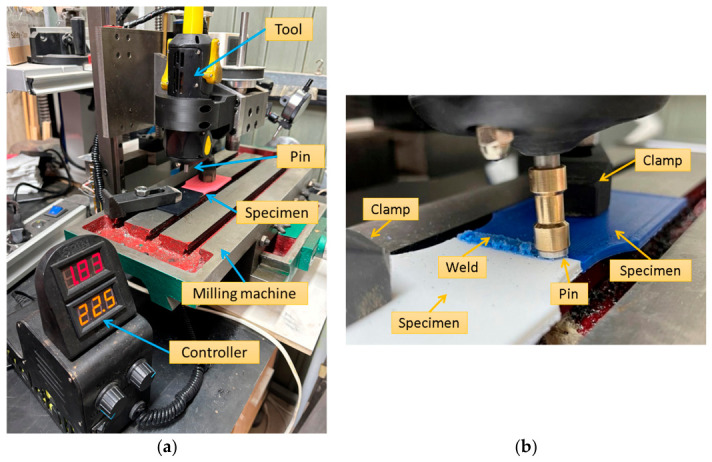
(**a**) Friction Stir Welding Equipment mounted on a CNC machine; (**b**) welding setup and specimen fixation.

The experimental framework employed a propotype of the patented portable FSW device (ES2952553) [37], consisting of a handheld tool unit and an external control box for independent adjustment of processing parameters. To ensure precision, repeatability, and minimization of operator-induced variability, the device was securely mounted on a purpose-built vertical milling machine equipped with Cartesian axes. This configuration enabled accurate control of the tool vertical position (*z*-axis), tilt angle, and welding traverse speed, while rotational speed and filler feed rate were regulated through the integrated device controller. The experimental setup (Figure 1a) allowed systematic investigation of the influence of process parameters on weld penetration, material flow behavior, transient and steady-state zone formation, and the resulting mechanical performance of additively manufactured poly(lactic acid) (PLA) specimens with different infill densities.

Other key operational parameters, such as the rotational speed of the tool head and the feed rate of the filler material, were regulated directly through the integrated controller of the FSW device itself. This hybrid configuration, combining manual guidance with CNC-like precision in selected axes, proved particularly advantageous for conducting controlled research trials on heat-sensitive polymeric materials, allowing reliable evaluation of how variations in tilt angle (0° or 8°), welding speed (10 mm/min or 20 mm/min), rotational speed (1500–2500 rpm), and material addition influence weld quality. The 8° tilt angle was selected based on preliminary trials to compensate for the lower stiffness and higher compliance of polymer substrates compared to metallic systems, thereby improving forging pressure and material consolidation. This configuration allowed systematic analysis of penetration depth, formation of transient versus steady-state zones, flash generation, material mixing, and the resulting mechanical performance of the joints in additively manufactured PLA specimens with different infill densities.

The welding process was conducted using a single-square-groove butt joint configuration with no edge preparation. The specimens were aligned on a flat steel reference surface to ensure planarity, maintaining direct contact at the joint interface. Mechanical stability during the process was ensured by securing both halves to the worktable using bolted clamping fixtures, which prevented any thermal distortion or lateral displacement (Figure 1b). The experimental setup enabled precise and repeatable control over key process variables, including the tool’s rotational speed, tilt angle, and traverse (welding) speed. In configurations involving material addition, the portable FSW device incorporated a 3 mm diameter PLA rod as the filler material. This filler rod was continuously fed through a dedicated central hole in the rotating welding head at a programmable feed rate, while the operator manually guided the tool along the butt joint interface to produce the weld bead.

**Figure 2:**
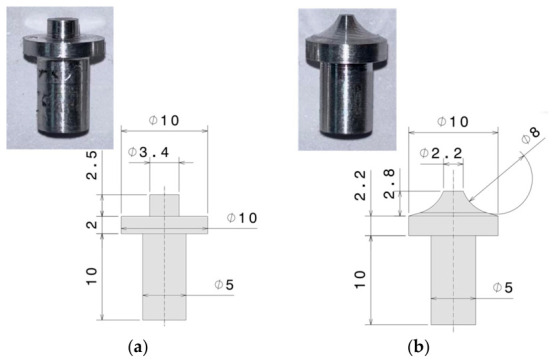
Geometries and dimensions of: (**a**) cylindrical pin (tool 1) and (**b**) truncated conical pin (tool 2), respectively.

Two distinct tool geometries were evaluated (see Figure 2):

- Tool 1 (Figure 2a). A cylindrical pin with a constant diameter of 3.4 mm and 2.5 mm height, and a shoulder with a diameter of 10 mm.
- Tool 2 (Figure 2b). A truncated conical tapered pin with a tip diameter of 2.2 mm that gradually widened to a 10 mm diameter at the base (shoulder region).

Both tools were manufactured from 6082-T6 aluminum alloy, selected for its good thermal conductivity and sufficient mechanical strength during friction-based processing of thermoplastics. The modular design made it possible to directly compare the effect of pin geometry on material flow, heat input, weld penetration, flash formation, and overall joint performance. The cylindrical pin tended to generate a more uniform shear distribution across the weld zone. In contrast, the truncated conical pin promoted a more gradual plastic deformation of the material and, in some cases, improved mixing near the weld root, likely due to the progressive increase in contact pressure toward the upper surface. However, previous studies do not clearly establish the superiority of one geometry over the other, as the results strongly depend on the specific processing conditions.

**Figure 3:**
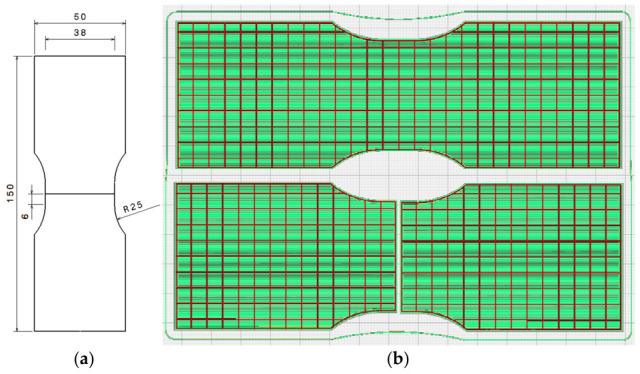
(**a**) Specimen geometry dimensions; (**b**) Slicing stage prior to printing the 15% infill specimens, highlighting the 0–90° grid for welded (top) and unwelded specimens (bottom).

The fabrication parameters for the PLA specimens were set in accordance with the filament supplier’s recommended guidelines and the prescribed specimen geometry outlined in the AWS B4.0:2016 standard [38]. All test pieces were produced using Fused Deposition Modeling (FDM) technology and positioned on the build plate as illustrated in Figure 3b. The PLA used in this study was supplied by Smart Materials 3D (Alcalá la Real, Spain) [39]. Printing was performed on a delta-architecture 3D printer, specifically the Anycubic Kossel Linear Plus model manufactured by ANYCUBIC Technology Co., Ltd. (Shenzhen, China).

### 2.1. Manufacturing Parameters

Two internal infill levels were selected: 15% and 100%, both using a 0–90° grid pattern. All specimens had a uniform thickness of 3 mm, and the butt-joint edges were carefully smoothed and squared to ensure proper alignment and intimate contact during welding. Throughout the welding trials, the tool temperature was periodically monitored with a handheld Fluke 566 infrared thermometer (Fluke Corporation, Everett, WA, USA) to obtain qualitative information on the thermal conditions at the shoulder and pin. By varying only the infill percentage, a direct comparison was possible between sparsely filled structures, representative of lightweight AM designs, and fully dense specimens, which more closely resemble solid injection-molded parts. All other printing parameters were kept constant, as shown in Table 1. The remaining parameters were selected based on previous experimental findings [40]. The specimen geometry (Figure 3a) followed the AWS B4.0:2016 standard [38], as the samples were intended for tensile testing and subsequent mechanical evaluation. Figure 3b illustrates the slicing stage prior to printing the 15% infill specimens (both complete and split configurations), highlighting the 0–90° grid configuration of the internal structure in the unwelded samples. The specimens intended for welding followed the same internal configuration but were initially printed as two separate halves. The use of a 0–90° grid orientation, rather than the mechanically optimal ±45° pattern, was intentionally chosen to facilitate the visual and microstructural analysis of material flow and defect formation during FSW, in line with previous polymer welding studies [27,41].

The welding tests were organized into four groups. Groups P1 and P2 were designed to evaluate which welding speed yields the best results. They used 15% infill specimens welded at 10 mm/min and 20 mm/min, respectively, with an 8° tool inclination and 1950 rpm. In these cases, the added material acted as the stirring medium instead of a conventional FSW pin. Groups P3 and P4 were designed to evaluate the effect of pin geometry on weld quality, using specimens printed with 100% infill and welded with cylindrical and truncated conical tool pins, respectively. For these groups, the welding speed was set to 20 mm/min, based on previous experience [40], and the tool rotational speed was fixed at 2500 rpm.

Two additional groups of specimens were printed as single, solid pieces to evaluate their mechanical properties as a reference for the welded samples. One group, designated S15, had 15% infill, while the other, S100, was fully dense with 100% infill. In each configuration, five specimens were tested.

**Figure 4:**
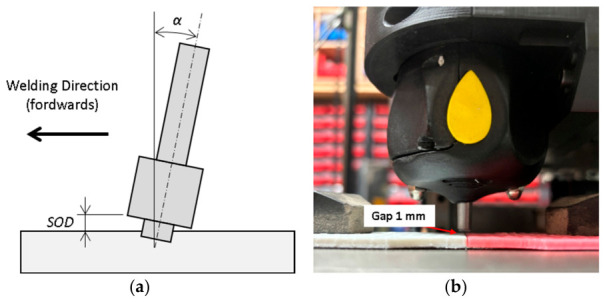
(**a**) Tool positioning relative to the material to be welded, indicating the forward welding direction, stand-off distance (SOD), and tilt angle; (**b**) gap between the rotating support and the specimen for 15% infill specimens.

During the welding process, the Stand-off distance (SOD) was adjusted to 1 mm, according to reported analyses [42], as shown in Figure 4a, for 100% infill specimens. For 15% infill specimens, without pin, the separation between the tool and the specimen was 1 mm, as illustrated in Figure 4b.

### 2.2. Welds Evaluation

To evaluate the visual weld quality, the optical method based on bands or lines is employed, a widely used approach for analyzing the quality of friction welds [43,44,45,46].

**Figure 5:**
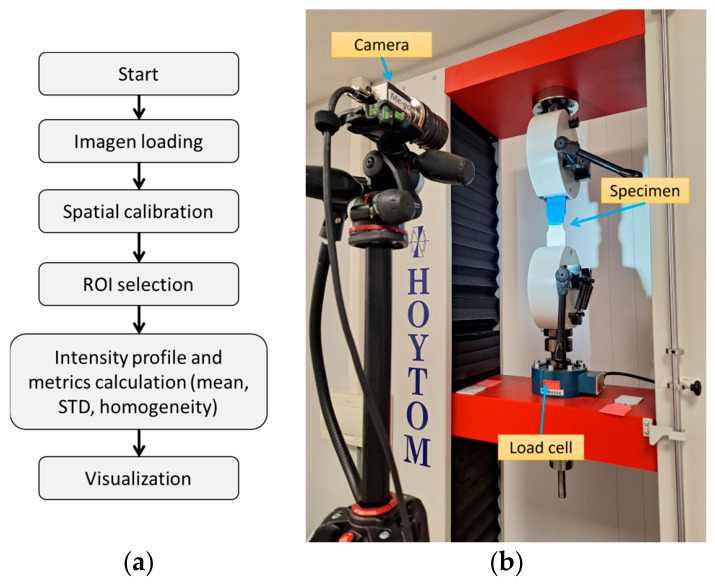
(**a**) Flowchart of the band method to analyze the weld quality; (**b**) setup for the tensile tests using DIC.

The procedure (Figure 5a) consisted of loading and preprocessing the weld image (grayscale conversion and normalization), performing manual spatial calibration using a 38 mm distance (reference distance in the narrowest area), selecting a rectangular ROI over the weld zone, extracting the longitudinal intensity profile (mean and standard deviation per column), converting the axis to real distance (mm), computing the profile gradient, and deriving key metrics such as mean intensity and homogeneity index, according to Equation (1), where σ is the intensity standard deviation and μ is the average intensity. Results were visualized through the plot of the shaded standard deviation and the gradient along the analyzed length. Weld thickness was evaluated using profile projector images by performing nine equidistant measurements across the sample width. This approach allowed for the calculation of both the average thickness and the dispersion for each tested group.

(1) HI=1−σImean

Finally, the mechanical behavior is evaluated through the tensile test of the welded and unwelded samples. Tensile tests were performed using an MTS C43 universal testing machine (Hoytom S.L., Leioa, Bizkaia, Spain) at a crosshead speed of 5 mm/min, in accordance with ASTM D638 [47]. Digital Image Correlation (DIC) was employed to evaluate the displacement field and subsequently calculate strain during loading. Image acquisition was carried out using a 5 MP ME-go camera (MEgo GmbH, Hilden, Germany) operating at 62 fps. DIC analysis was conducted using GOM Correlate software 2019 (GOM GmbH, Braunschweig, Lower Saxony, Germany). Post-test, fracture surfaces were analyzed via profile analyzer TESA VISIO (TESA Technology, Renens, Vaud, Switzerland) to identify failure modes such as ductile fibrillation or brittle cleavage. The complete setup is shown in Figure 5b.

## 3. Results and Discussion

This section presents the main findings from the welding tests. The analysis begins by addressing the presence of entry and exit burrs, the degree of weld penetration, and the variation in specimen mass. It then describes the different zones identified in the welds, distinguishing between stable and transient regions. Images of the welded specimens are included to illustrate the observations.

### 3.1. Tensile Tests

**Figure 6:**
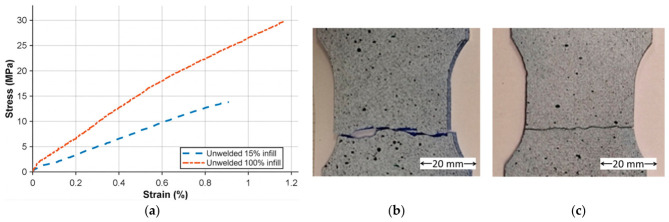
(**a**) Stress–strain curves of unwelded 3D-printed specimens with 15% and 100% infill densities; (**b**) failure of 100% infill specimen; (**c**) failure of 15% infill specimen.

The results of the tensile tests carried out on the unwelded specimens are shown in Figure 6a. A clear difference can be observed between the samples manufactured with 15% infill and those produced with 100% infill. The maximum tensile strength recorded for the 100% infill specimens was 29.2 ± 1.0 MPa and the strains were 1.19 ± 0.15%, while for the 15% infill specimens, the values were 13.8 ± 1.7 MPa and 0.91 ± 0.12%.

In terms of stiffness, the elastic modulus of the 15% infill samples was 1.35 ± 0.25 GPa, whereas the 100% infill specimens reached 2.58 ± 0.1 GPa. Regarding elongation at break, the lower infill specimens exhibited a more brittle behavior. Figure 6b and Figure 6c show the specimens after testing, for 100% and 15% infill, respectively. Overall, these results are consistent with values previously reported in the literature [48].

For welded specimens, the experimental data in Table 2 indicates that the infill density of the 3D-printed substrates is the primary determinant of the joint’s mechanical integrity. For specimens P1 and P2, which feature a 15% infill, the resulting stress values remain relatively low, ranging from 4.16 ± 0.12 to 3.67 ± 0.05 MPa. This indicates that the internal porosity reduces effective load transfer area and limits thermo-mechanical consolidation during FSW. A lower welding speed of 10 mm/min in P1 provided a marginal increase in strength over P2, suggesting that longer interaction time slightly improves consolidation in porous substrates.

In contrast, transitioning to 100% infill specimens (P3 and P4) significantly enhances the load-bearing capacity of the welds, with stress values peaking at 8.02 ± 1.42 MPa. These values are consistent with previously reported FSW strengths in thermoplastic systems, although lower than bulk PLA tensile strength due to localized heat-affected softening. The corresponding joint efficiency, calculated relative to the tensile strength of the 100% infill base material, ranges between approximately 21% and 27%, which is consistent with portable or non-industrial FSW configurations reported for thermoplastic systems. This improvement is largely attributed to the introduction of a tool pin and an increased rotation speed of 2500 rpm. The cylindrical tool pin used in P3 outperformed the truncated conical geometry of P4, generating more uniform shear deformation and improved interlayer mixing. The data suggest that, for fully dense printed parts, the cylindrical profile is more effective at reducing interfacial voids and enhancing load transfer across the weld interface.

The strain at break of all welded specimens, averaging between 0.40% and 0.45%, indicates a relatively stable brittle behavior regardless of the infill or tool geometry. However, the higher standard deviations observed in the stress results for P3 and P4 point to a greater sensitivity to localized thermal gradients and material flow patterns in high-density parts. These values are significatively lower than that of the base material, indicating an embrittlement of the weld zone, also reported by Derazkola et al. [49] and associated with molecular and thermal degradation, or incomplete reconsolidation during cooling.

**Figure 7:**
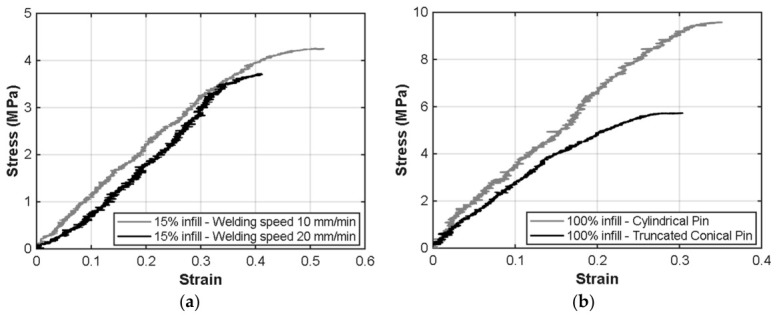
(**a**) Stress–strain curves of specimens with 15% and (**b**) 100% infill densities.

Figure 7a,b show representative specimens for 15% and 100% infill. At 15% infill, stiffness is similar, though maximum stress is higher at 10 mm/min welding speed. At 100% infill, differences are more pronounced, with the cylindrical pin yielding a higher elastic modulus and greater strength. The reported stress values should be regarded as nominal, as the effective cross-sectional area at the weld interface may differ from that in the base material.

### 3.2. Visual Inspection

**Figure 8:**
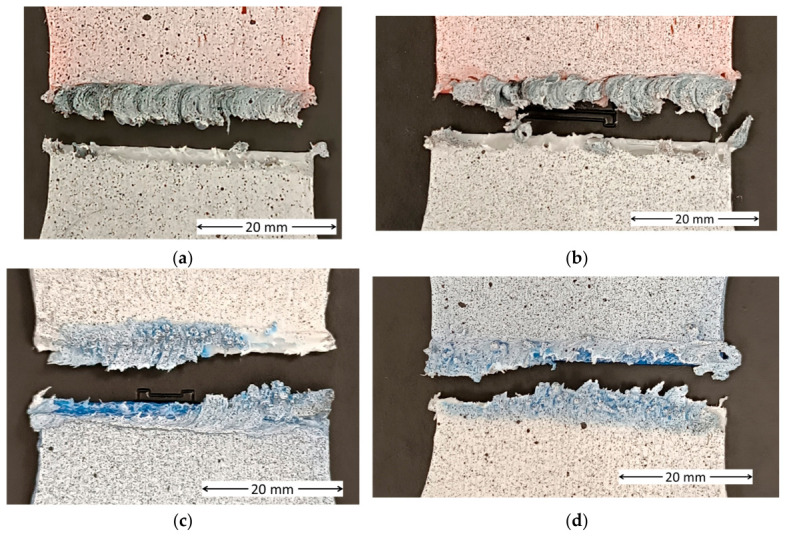
Fracture mode of (**a**) 15% infill, 10 mm/min; (**b**) 15% infill, 20 mm/min; (**c**) 100% infill, cylindrical tool; and (**d**) 100% infill, conical tool.

Regarding the failure modes, post-fracture analysis shows that in specimens with 15% infill, the weld has been completely detached from the base material (Figure 8a,b), with the fracture occurring at the weld–substrate interface. This indicates that, although the material was properly stirred, it was not fully consolidated into the voids of the porous substrate. Since the welding occurred primarily at the front face, this region remains weakened, and stress concentration leads to premature failure. This behaviour is consistent with findings by Arif et al. [50], who observed that in polymer FSW, defects tend to localize on the retreating side due to asymmetric heat distribution, unlike metallic FSW where they typically form on the advancing side. A similar mechanism has been reported in FSW of polymers, where insufficient heat input prevents adequate molecular interdiffusion, resulting in weak bonding and interfacial failure [49,51].

In specimens with 100% infill, fracture propagated through the weld zone (Figure 8b,c), covering a significant portion of its width. Unlike the 15% infill specimens, complete weld detachment is not observed here, suggesting better material consolidation owing to the denser substrate. This indicates that the weld zone remains the weakest region under tensile loading. This type of through-weld fracture has been widely reported in polymer FSW and is generally attributed to microstructural changes and residual porosity within the stir zone [52]. As no additional material was added, a slight depression forms at the weld surface, acting as a geometric stress concentrator that promotes crack initiation. These results are consistent with observations by Derazkola et al. [49], who identified surface shrinkage features as preferential crack initiation sites in FSW of polymers.

Furthermore, the fractures shown in Figure 8 are consistent with the aforementioned embrittlement. This brittle behavior is further supported by the tensile results, where the welded specimens exhibited significantly lower strain-at-break values compared to their unwelded counterparts, aligning with the lack of visible plastic deformation in the images.

**Figure 9:**
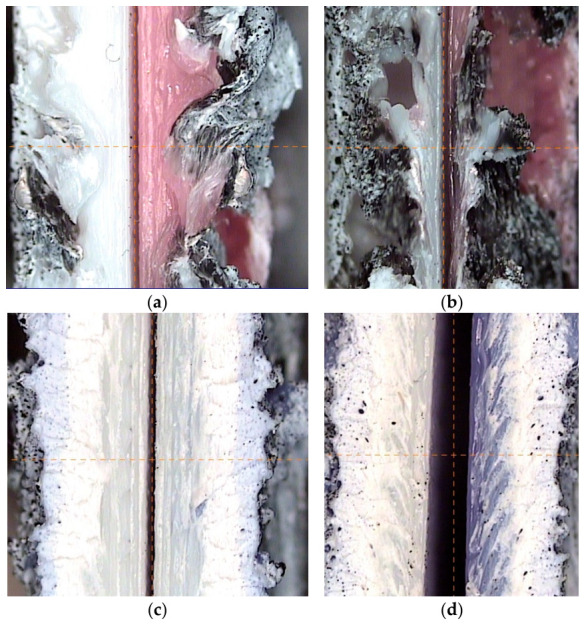
Detailed inspection of the fractured faces for (**a**) 15% infill, 10 mm/min; (**b**) 15% infill, 20 mm/min; (**c**) 100% infill, cylindrical tool; and (**d**) 100% infill, conical tool.

The images obtained from the profile projector allow a detailed examination of the generated fractures. In specimens P1 and P2 (Figure 9a,b), the bonded regions are very limited, confined to a narrow band with numerous defects. In P1 (Figure 9a), corresponding to the lower welding speed, a slight increase in weld thickness is observed, which corroborates the mechanical test results.

For specimens with 100% infill (Figure 9c,d), the contact area is much larger than in the previous cases, resulting in improved mechanical properties. In the case of the cylindrical pin (Figure 9c), weld penetration is greater than that achieved with the conical pin (Figure 9d). However, the conical pin exhibits a herringbone-like pattern due to its taper, which produces more stirring. While this would be expected to enhance mechanical properties, the effect is offset by the reduced weld penetration. Consequently, it can be concluded that the cylindrical pin, with its larger upper diameter of 3.4 mm, generated a greater thermal effect, producing fusion over a larger volume, despite being shorter than the conical pin.

Weld thickness results from the cross-sections in Figure 9 correlate with the mechanical properties. For the 15% infill, weld thicknesses were 1.41 ± 0.37 mm at 10 mm/min and 1.28 ± 0.41 mm at 20 mm/min. For the 100% infill, the cylindrical and conical pins yielded 2.42 ± 0.39 mm and 2.33 ± 0.64 mm, respectively.

Comparison with tensile test data shows that for 15% infill, higher weld thickness at low speed resulted in a higher tensile strength (4.16 ± 0.12 MPa) compared to high speed (3.67 ± 0.05 MPa). Furthermore, the cylindrical pin showed lower thickness dispersion and greater homogeneity. This led to a tensile strength of 8.02 ± 1.42 MPa, surpassing the 6.25 ± 1.43 MPa of the conical pin, which exhibited higher thickness variability.

**Figure 10:**
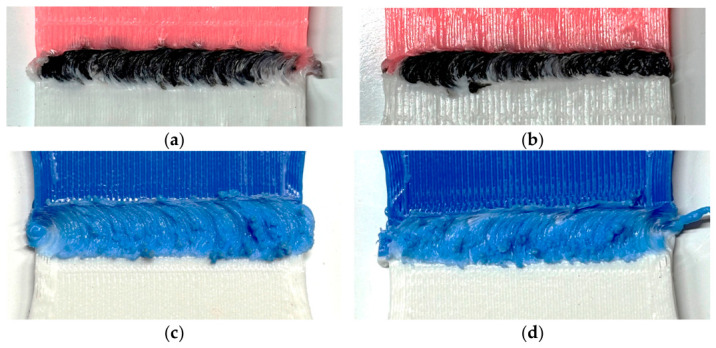
Details of the welds analyzed optically: (**a**) 15% infill, 10 mm/min; (**b**) 15% infill, 20 mm/min; (**c**) 100% infill, cylindrical tool; and (**d**) 100% infill, conical tool.

**Figure 11:**
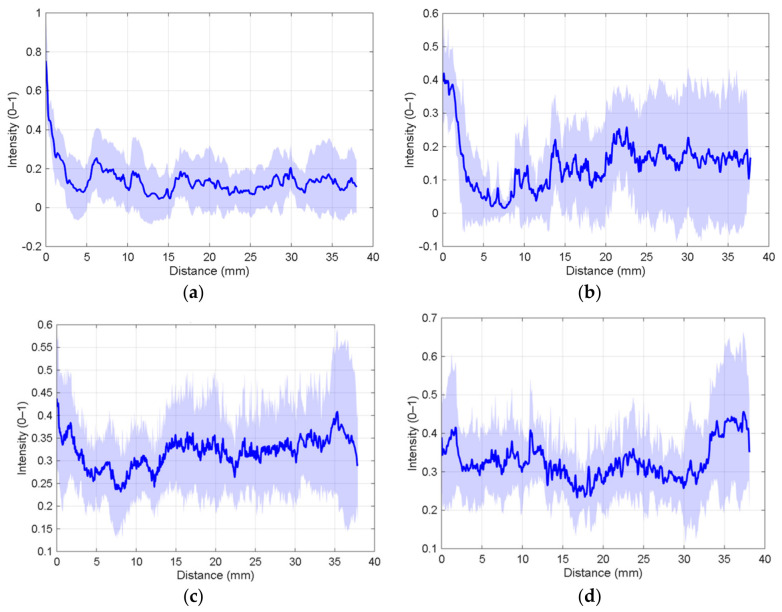
Mean profile (dark blue) and standard deviation (clear blue) from optical evaluation: (**a**) 15% infill, 10 mm/min; (**b**) 15% infill, 20 mm/min; (**c**) 100% infill, cylindrical tool; and (**d**) 100% infill, conical tool.

The band method was applied to representative specimens of each group (in order P1, P2 P3 and P4) as shown in Figure 10a–d. The integration of optical band method profiles with mechanical tensile data reveals a clear divergence in joint quality based on infill density and tool configuration (Figure 11a–d). Specimens P1 and P2 (15% infill) exhibited a characteristic sharp drop in intensity at the beginning of the profile, followed by significant fluctuations after the 10 mm mark. This erratic behavior indicates that the internal voids of the 15% infill fail to provide a stable substrate for the tool shoulder, leading to inconsistent plastic deformation. Furthermore, the high dispersion observed in P2 (eb), particularly toward the end of the weld, suggests a lack of process repeatability likely tied to localized thermal gradients and the uneven distribution of the filler material. Without a tool pin to drive volumetric mixing, the filler material serves as a non-structural deposit, resulting in poor consolidation and a baseline tensile strength of approximately 4 MPa. In contrast, the 100% infill specimens (P3 and P4) demonstrated significantly higher mean intensity values and superior structural homogeneity (Figure 11c,d). The absence of internal voids allowed the tool shoulder to exert the necessary forging pressure to generate a solid heat-affected zone, directly correlating to the observed increase in tensile resistance. P3, processed with a cylindrical pin, showed the most consistent optical profile and achieved the peak tensile stress of 8.02 ± 1.42 MPa, suggesting that the cylindrical geometry optimizes material flow and pressure distribution in solid substrates. While P4 (truncated conical pin) displayed greater fluctuations and dispersion at the weld edges, the overall trend confirms that full infill density facilitates a more uniform thermal and mechanical environment. Ultimately, these results highlight that while filler material is used in low-infill joints to compensate for mass loss, it cannot replicate the microstructural uniformity achieved in solid joints processed with a tool pin. The stability of the optical intensity in P3 and P4 confirms that a solid substrate allows for constant heat generation and forging pressure, which are fundamental requirements for high-performance friction stir welded joints in additive manufacturing.

Table 3 summarizes the results of the optical analysis of the welds.

### 3.3. Engineering Implications for Portable FSW of PLA

The experimental results indicate that substrate infill density is the most critical parameter governing joint performance when applying portable FSW to FDM-printed PLA structures. Low-density configurations (15% infill) exhibit limited consolidation due to internal porosity, resulting in poor load transfer and premature interfacial failure, even when filler material is supplied. This is consistent with Anaç et al. [36], who reported that at low infill ratios (20% and 40%) the weld quality was negatively affected. Therefore, portable FSW is better suited to fully dense or near-solid substrates when structural integrity is required.

Tool pin geometry represents the second key parameter influencing weld quality. The cylindrical pin configuration demonstrated superior tensile performance and more uniform material flow compared to the truncated conical design, suggesting that enhanced shear deformation and stable pressure distribution improve consolidation efficiency. These findings align with other researchers [53,54], who demonstrated that smooth-profile pins produce fewer internal voids than sharp-edged geometries, which tend to induce over-stirring and defects. For practical applications, the use of a cylindrical pin combined with adequate rotational speed is recommended to ensure consistent plasticization and defect minimization. Conversely, insufficient substrate density or the absence of a tool pin significantly reduces joint efficiency and should be avoided in load-bearing applications.

While the present results demonstrate the feasibility and mechanical effectiveness of portable FSW under quasi-static tensile loading, it should be noted that the current study is limited to monotonic tensile performance. Fatigue behavior and long-term thermal aging effects remain to be investigated to fully assess the structural reliability of welded PLA components.

## 4. Conclusions

The results confirm that a portable Friction Stir Welding (FSW) tool can effectively join additive-manufactured PLA parts. Specimens with 15% infill showed limited weld penetration and poor consolidation, leading to low tensile strength of 3.67–4.16 MPa (CV = 3–5%) and failure at the weld–substrate interface. Lower welding speeds reported slightly better results in terms of strength. Optical analysis revealed erratic intensity profiles and low homogeneity (index < 0.02), highlighting the instability of the joint. For such low-density materials, adding filler or adjusting process parameters is necessary to achieve satisfactory bonding.

In contrast, specimens with 100% infill exhibited much higher mechanical performance, with tensile strength ranging from 6.25 to 8.02 MPa and lower relative variation (CV = 10–18%). Fractures propagated through the weld without detachment, indicating that failure occurred within the weld rather than at the interface. Among these, the cylindrical pin produced slightly deeper and more uniform penetration, more uniform optical profiles, and higher tensile strength 8.02 ± 1.42 MPa (CV = 17.7%) than the truncated conical pin 6.25 ± 1.43 MPa (CV = 22.9%). Although the conical pin improved surface mixing through a herringbone stirring pattern, its lower penetration and higher variability in penetration depth limited overall mechanical performance.

The combined analysis of optical profiles and tensile data confirms that both infill density and tool geometry are key factors in joint quality. Fully dense specimens achieve stable heat generation and consistent material flow, making additional filler unnecessary, while low-infill parts require extra material to compensate for porosity. Overall, these findings demonstrate that FSW is a reliable method for joining PLA AM parts and that combining solid substrates with an appropriately designed tool pin is essential to maximize strength, consistency, and weld integrity. Future work should include detailed thermal characterization of the weld zone and long-term durability assessment under cyclic loading conditions to further optimize portable FSW of FDM-printed poly(lactic acid) structures.

## 5. Patents

The novel Friction Stir Welding (FSW) device is protected by patent ES2952553.

## Figures and Tables

**Table 1:** Printing parameters. Parameter categories are in bold, followed by their respective values for 15% and 100% infill.

Parameter	15% Infill	100% Infill	Parameter	15% Infill	100% Infill
**Main deposition parameters**			**Speed**		
Layer height (mm)	0.2		Print speed (mm/s)	60	
Wall thickness (mm)	0.8		Travel speed (mm/s)	120	
Initial layer thickness (mm)	0.3		Infill speed (mm/s)	60	
Nozzle diameter (mm)	0.4		Top/bottom layer speed (mm/s)	30	
Bed temperature	40		Outer wall speed (mm/s)	40	
Nozzle temperature	215		Inner wall speed (mm/s)	60	
**Support**			**Infill**		
Support	No		Solid top infill	Yes	
Skirt	Yes		Solid bottom infill	Yes	
Skirt thickness (mm)	2		Infill density (%)	15	100
**Other**			Infill pattern	Grid	
Enable cooling fan	Yes		Top/bottom thickness (mm)	0.6	
Extrusion expansion (%)	100		Infill direction (°)	0	
Extrusion overlaps (mm)	0.15		Top/bottom infill direction (°)	0	

**Table 2:** Summary of variables and results in the welding of test specimens.

	Rotation Speed (rpm)	Welding Speed (mm/min)	Material Feed (mm/min)	Strain at Break (%)	Tensile Strength (MPa)
**P1. 15% infill. Without tool pin. Low welding speed**	1950	10	20	0.45 ± 0.05	4.16 ± 0.12
**P2. 15% infill. Without tool pin. High welding speed**	1950	20	20	0.44 ± 0.05	3.67 ± 0.05
**P3. 100% infill. Cylindrical tool pin. No material addition**	2500	20	None	0.45 ± 0.09	8.02 ± 1.42
**P4. 100% infill. Truncated conical pin. No material addition**	2500	20	None	0.40 ± 0.08	6.25 ± 1.43

**Table 3:** Summary of the results of the optical analysis.

Sample	Analyzed Length (mm)	Mean Intensity	Mean Std. Deviation	Homogeneity Index
P1	37.98	0.1385	0.1364	0.0157
P2	37.77	0.466	0.1448	0.0121
P3	37.90	0.3190	0.1039	0.6744
P4	38.09	0.3261	0.1061	0.6745

## Data Availability

The original contributions presented in this study are included in the article. Further inquiries can be directed to the corresponding author.
